# Ultra-Wideband Radar-Based Sensing Poultry Litter Moisture Content Monitoring System

**DOI:** 10.3390/ani16091382

**Published:** 2026-04-30

**Authors:** Haotang Li, Zhenyu Qi, Tanvir Ahmed, Shungeng Zhang, Sen He, Zi Wang, Guoming Li

**Affiliations:** 1 Department of Electrical and Computer Engineering, University of Arizona, Tucson, AZ 85721, USA; haotangl@arizona.edu (H.L.); qzydustin@arizona.edu (Z.Q.); senhe@arizona.edu (S.H.); 2 School of Computer and Cyber Sciences, Augusta University, Augusta, GA 30912, USA; taahmed@augusta.edu (T.A.); szhang2@augusta.edu (S.Z.); 3 Department of Poultry Science, University of Georgia, Athens, GA 30602, USA; gmli@uga.edu

**Keywords:** ultra-wideband radar, litter moisture content (LMC), non-contact sensing, precision livestock farming, bedding quality, sensor characterization

## Abstract

High litter moisture content (LMC) in poultry houses is a primary driver of footpad dermatitis, elevated ammonia emissions, and bacterial proliferation. These conditions directly compromise broiler welfare and productivity. Existing monitoring methods, including oven-drying, contact-based sensors, and near-infrared spectroscopy, suffer from invasiveness, single-point limitation, or surface-only measurement. This study investigates ultra-wideband (UWB) impulse radar as a non-contact sensing modality for estimating the LMC of cedar wood shaving bedding under controlled laboratory conditions. A four-phase experimental program was conducted. Phases 1–3 characterized signal–moisture relationships across 0–50% LMC, manure simulant contamination, and bedding structural changes (loose, compacted, caked). Phase 4 tested whether UWB radar can estimate litter LMC when a stationary broiler body obstructs the beam under combined contamination and structural conditions. A progressive feature engineering approach and an SVC-gated mixture-of-experts regression architecture were used to address each confounding factor. Full technical details are provided in the Methods Section. Under clean conditions, the baseline model achieved $R^{2}=0.97$ and RMSE = 2.48% LMC. Under combined realistic conditions (manure contamination, caked bedding, centered carcass), the full pipeline achieved $R^{2}=0.91$ and RMSE = 4.53% LMC, with 98.8% bird detection accuracy from the radar signal alone. These laboratory findings suggest that the UWB radar can sense litter moisture through a stationary broiler body. The results support its potential as the sensing core of a non-contact monitoring system for precision poultry farming.

## Simple Summary

High moisture in poultry litter causes welfare problems such as footpad dermatitis and ammonia burns. Current monitoring methods are either destructive or limited to surface measurements. This study tests ultra-wideband (UWB) radar as a non-contact method to estimate litter moisture content (LMC) under laboratory conditions, examining the effects of manure contamination, bedding compaction, and bird-body obstruction on sensing performance.

## 1. Introduction

Global broiler meat production has grown steadily over the past two decades, reaching approximately 100 million tonnes per year [1]. At the same time, public awareness and regulatory pressure on animal welfare have increased considerably [2]. In modern floor-raised housing systems, bedding material is the primary interface between the bird and its environment. The condition of this litter directly affects bird health, behavior, and welfare [3,4]. Excessive litter moisture is one of the most consequential management challenges in impacting litter condition. When litter moisture content (LMC) exceeds approximately 25–30%, the risk of footpad dermatitis (FPD) increases sharply [3,5]. The FPD causes pain, reduces locomotion, and is widely used as an on-farm welfare compliance indicator in the European Union [6]. High LMC also accelerates microbial decomposition of uric acid, releasing ammonia into the house atmosphere [7]. Elevated ammonia concentrations damage the respiratory tracts and eyes of both birds and farm workers [8]. Wet litter also provides a favorable environment for pathogenic bacteria, including *Salmonella* and *Campylobacter*, posing food safety risks [9].

Several factors contribute to litter moisture accumulation during the grow-out cycle. Drinker spillage, fecal excretion, and inadequate ventilation are the primary sources [10]. As birds grow, stocking density effectively increases, adding more excreta per unit area. Over time, fresh loose wood shavings become compacted and eventually form a caked surface layer [11]. Moisture distribution is also spatially heterogeneous. Areas near drinker lines and along house walls tend to be wetter than central zones [3]. These spatial and temporal variations along with infrequent manual inspection make LMC difficult to monitor and manage. A continuous, spatially resolved monitoring system would enable earlier intervention and more targeted management [12].

Existing LMC monitoring methods fall into three groups, each with intrinsic limitations. The gravimetric reference, oven-drying at 105 °C for 24 h [13,14], is accurate but destructive and non-continuous. Contact-based capacitive and resistive probes [7,15] provide only single-point readings, must be buried in the litter, and suffer from manure fouling and corrosion [11]. Optical and spectroscopic methods such as near-infrared (NIR) reflectance [15,16] and hyperspectral imaging [17] are non-contact but limited to 1–2 mm of surface penetration [18], leaving subsurface moisture beneath caked crusts invisible. No existing method simultaneously provides non-contact operation, subsurface penetration, real-time capability, and robustness to commercial poultry house conditions. This gap motivates the search for an alternative sensing modality.

Ultra-wideband (UWB) radar is a promising candidate for addressing these limitations. In brief, UWB radar transmits very short, low-power pulses (bandwidth > 500 MHz, typically in the 3.1–10.6 GHz band [19]) that yield fine range resolution and can propagate through most non-metallic materials. Combined with compact hardware and tolerance to dust and varying lighting, these properties make UWB well-suited to agricultural environments such as poultry houses [20].

UWB-based sensing has been applied to moisture measurement in several related domains. In soil science, ground-penetrating radar systems operating at UWB frequencies are routinely used to estimate soil water content [21,22]. In civil engineering, UWB radar has been used to detect moisture in concrete, brick, and timber structures [23]. In food science, similar techniques have been explored for measuring moisture in grain and other granular materials [24]. The physical basis is straightforward. Water has a high relative permittivity ($\varepsilon_{r}\approx 80$), while dry organic materials such as wood shavings exhibit much lower values ($\varepsilon_{r}\approx 2$–5). This large dielectric contrast produces measurable changes in signal amplitude, propagation delay, and frequency content as moisture increases [25].

Despite this body of related work, to the best of our knowledge, UWB radar has not previously been investigated for poultry litter moisture content monitoring. No prior study has examined whether it can operate under the combined effects of manure contamination, structural changes in the bedding, and obstruction by the bird body. This is the gap that the present manuscript addresses. Poultry litter presents unique challenges that are absent in cleaner substrates. Manure contamination introduces additional ionic and organic content that may alter the dielectric response independently of water content [26]. Structural transitions from loose to compacted and caked states change the material density and pore structure, affecting electromagnetic wave propagation [11]. In a real poultry house, the birds themselves are positioned above the litter and may scatter or attenuate the radar signal. No prior study has characterized how these factors affect UWB radar performance in this specific application, or whether a radar-based system can function reliably despite them.

This study investigates UWB impulse radar as the sensing core of a non-contact poultry litter monitoring system. A four-phase experimental program was designed to isolate individual confounding factors progressively. To the best of our knowledge, this is the first study to investigate UWB radar for poultry LMC monitoring. It is also the first to address biological obstruction as a confounding factor in radar-based moisture sensing. The specific contributions are as follows:

1. We establish the baseline relationship between UWB channel impulse response (CIR) features and LMC across the 0–50% range. We compare five regression algorithms (Ridge, SVR, LightGBM, XGBoost, Gaussian Process Regression) on an initial 16-dimensional feature vector to identify the most suitable base model for this sensing task.
2. We quantify how manure contamination and bedding structural changes (compaction, caking) degrade estimation accuracy. We introduce a progressive feature engineering approach in which physically motivated features are added at each phase, expanding the feature vector from 16 to 64 dimensions. We also demonstrate that UWB radar can detect subsurface moisture beneath a caked surface layer, a capability unavailable to surface-only methods.
3. We demonstrate, for the first time, that UWB radar can estimate LMC when a broiler body obstructs the beam. We introduce spectral and MIMO cross-channel features that characterize the body’s scattering signature and an SVC-based scene classifier that jointly detects bird presence and bedding structural state from the radar signal alone.
4. We develop an adaptive estimation pipeline through progressive model refinement tied to each experimental phase. The pipeline uses a support vector classifier (SVC) gated mixture-of-experts (MoE) architecture with scene-specific SVR experts. This pipeline outperforms a single global model, particularly under bird obstruction.

The remainder of this paper is organized as follows. Section 2 describes the materials including UWB radar hardware, bedding materials, and broiler carcass; the experimental setup, four-phase experimental design with progressive feature extraction and model development, and statistical analysis methods. Section 3 reports the results for each phase. Section 4 discusses the physical interpretation of the findings, practical implications for precision livestock farming, limitations of the laboratory study, and directions for future field validation. Section 5 summarizes the key conclusions.

## 2. Materials and Methods

This section first describes the sensing hardware, test materials, and experimental setup (Section 2.1, Section 2.2, Section 2.3, Section 2.4 and Section 2.5), then presents the four-phase experimental program in which the feature set and model architecture were developed progressively (Section 2.6, Section 2.7, Section 2.8 and Section 2.9), and concludes with the statistical analysis and validation procedures (Section 2.11).

### 2.1. UWB Radar System

**Figure 1:**
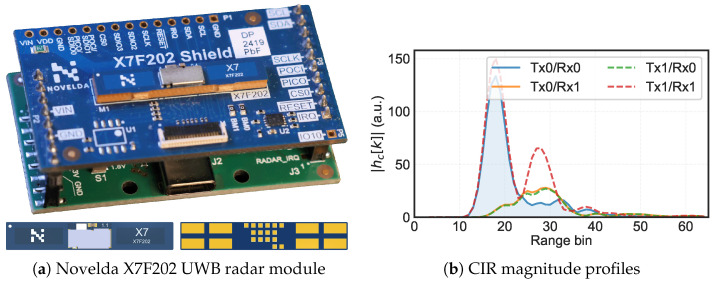
(**a**) The top view (upper) and front/back PCB photographs (lower) showing the dual Tx/Rx antenna layout. (**b**) CIR magnitude profiles ($|h_{c}\left[k\right]|$) across all four virtual channels for clean cedar shavings at 0% LMC and 50 cm antenna height (single frame). The Tx0/Rx0 channel (solid blue, shaded) serves as the primary channel. The peak amplitude ($A_{\mathrm{peak}}$) is annotated. Channel-dependent amplitude and multipath differences arise from the spatial separation of the Tx/Rx antenna pairs. All subsequent figures present results in terms of extracted features.

The UWB radar module used in this study was the X7F202 (Novelda AS, Oslo, Norway), a multichannel impulse-radar transceiver with a 7.875 GHz center frequency and 750 MHz (−10 dB) transmit bandwidth, qualifying as UWB under FCC Part 15 [19]. The module integrates two transmitters and two receivers, each with a PCB antenna (peak gain 4.14 dBi), producing four virtual channels (Tx0/Rx0, Tx0/Rx1, Tx1/Rx0, Tx1/Rx1) per measurement cycle and 64 complex-valued range bins per channel ($h_{c}\left[k\right]=I_{c}\left[k\right]+jQ_{c}\left[k\right]$). The module was operated at a 60 fps frame rate, with 2240 pulses integrated per frame and the transmit power set to the maximum trim level. Data were acquired via SPI on a Raspberry Pi running the Novelda RadarDirect SDK (pyx7configuration v0.6) under Python 3.10. Figure 1a shows the module hardware layout, the full set of SDK parameters is summarized in Table 1.

**Signal Acquisition and Preprocessing.** Each UWB measurement was recorded at 60 fps for 2 s, producing 120 frames per measurement and four CIR vectors per frame. Preprocessing removed the DC offset (by subtracting the mean complex value across all range bins), computed the magnitude profile $|h_{c}\left[k\right]|$ and power delay profile $|h_{c}{\left[k\right]|}^{2}$, estimated a noise floor from pre-reflection range bins, and computed the frequency-domain representation $H_{c}\left(f\right)=\mathrm{FFT}\left(h_{c}\left[k\right]\right)$. Features were extracted from each frame independently. Because the 120 frames per 2 s measurement are highly temporally correlated, cross-validation splits were performed at the measurement level rather than at the frame level: all frames from a given measurement were kept within the same fold (see Section 2.11). This grouping prevents leakage between training and validation sets and avoids overly optimistic performance estimates.

### 2.2. Bedding Material

Red cedar wood shavings were selected as the bedding material because cedar shavings are widely used in North American broiler production [11]. The product used was PetsPick Red Cedar Bedding (41 L compressed volume, kiln-dried, dust-free), purchased from Tractor Supply Co. (Brentwood, TN, USA). This product was recommended by a collaborating commercial poultry farm as representative of the bedding material used in their broiler houses. Although the shavings were kiln-dried by the manufacturer, fresh bedding can absorb ambient moisture during storage and handling. To confirm the baseline moisture level, three representative samples from the as-received bag were dried at 105 °C for 24 h following AOAC Method 930.15 [13]. The measured LMC of all three samples was below 1%, and the mean was 0.6%. This residual moisture was considered negligible relative to the 0–50% LMC range tested, and the as-received shavings were therefore assigned a nominal LMC of 0% for all subsequent experiments.

For each measurement, bedding was placed in a cardboard box lined with a polyethylene bag. The internal dimensions of the container were 30 cm × 25 cm × 12 cm (length × width × height). The bedding was filled to a depth of 10 cm, which is representative of typical litter depths in commercial broiler houses [11].

### 2.3. Manure Simulant

A synthetic manure simulant was prepared to introduce controlled contamination. This avoided the variability and biosafety concerns of real poultry manure. Synthetic fecal simulants are widely used in sanitation and sensor research because real excreta is pathogenic, malodorous, and highly variable between samples [27]. Most studies formulate a simulant to match the specific physical or chemical properties relevant to their measurement, rather than replicating the full composition [27]. In this study, the simulant was formulated to approximate the dielectric-relevant properties of fresh broiler excreta: moisture content, organic matter fraction, and ionic conductivity.

The simulant consisted of peat moss (40%), bentonite clay (15%), water (43%), and sodium chloride (2%) by mass. Each component was selected for a specific role in matching the dielectric behavior of real poultry excreta. Peat moss provided fibrous organic matter similar to undigested feed residue and cellulosic plant material present in poultry excreta. Its high water-holding capacity mimics the moisture retention behavior of real manure in litter. Bentonite clay contributed mineral content and viscosity. Its layered silicate structure has a high cation exchange capacity, which influences the low-frequency dielectric response of the mixture. Bentonite also gives the simulant a paste-like consistency similar to fresh excreta. Sodium chloride was added to approximate the ionic conductivity of poultry excreta. Poultry excreta has elevated electrical conductivity (EC) due to uric acid salts, dietary minerals, and electrolytes [26]. Maruthamuthu et al. [28] reported a mean EC of 5.74 dS/m for broiler litter collected from 110 commercial farms at the end of a six-week grow-out cycle, measured in a 1:10 litter-to-water suspension. Katuwal et al. [29] reported EC values of 4.5–7.2 dS/m for broiler litter from commercial facilities in the United States using the same 1:10 extraction method. These elevated EC values are attributed primarily to sodium, potassium, and ammonium ions concentrated through fecal and urinary excretion.

The NaCl content of the simulant (2% by total mass) was chosen to produce an ionic strength comparable to real broiler litter. The estimated bulk electrical conductivity of the formulated simulant was derived via a simplified Archie-type model that accounts for pore-water NaCl concentration, bound water fraction, and pore-network tortuosity. The resulting estimate falls in the single-digit dS/m range at the target LMC values and is consistent with the 4.5–7.2 dS/m reported for commercial broiler litter [28,29]. The full derivation is provided in Appendix C. The ionic conductivity of the simulant influences the UWB signal through dielectric loss. Higher conductivity raises the imaginary component of the complex permittivity $\varepsilon^{\prime \prime }$, attenuates the radar signal, and reduces the reflected power from the bedding layer [25].

### 2.4. Broiler Carcass

A commercially obtained whole broiler carcass (approximately 1.6 kg) was used as a proxy for a live bird in Phase 4. The use of a carcass is justified by the physics of electromagnetic interaction at UWB frequencies. At 7.875 GHz, the dielectric response of muscle tissue is dominated by the dipolar relaxation of water molecules, not by ionic conductivity [30]. Ionic conduction contributes primarily to the loss factor below approximately 1 GHz. Above this frequency, the water relaxation term ($\varepsilon^{\prime \prime }\propto \omega \tau /\left(1+\omega^{2}\tau^{2}\right)$) accounts for the majority of both the real and imaginary permittivity [30]. Because muscle tissue is approximately 75% water by mass, and this water content does not change in the hours following slaughter, the dielectric constant and loss factor at GHz frequencies remain stable postmortem.

Empirical data confirm this. Zhuang et al. [31] measured uncooked chicken breast tissue from 10 MHz to 1.8 GHz and found no significant difference in dielectric constant or loss factor between samples at 2 h and 24 h postmortem. Trabelsi [32] extended measurements to 20 GHz and confirmed that chicken tissue dielectric properties depend primarily on temperature and frequency. The small postmortem changes reported in the literature (5–15% conductivity decrease in the first hour) occur predominantly at frequencies below 2 GHz where ionic conduction dominates [30]. At 7.875 GHz, these ionic changes have a negligible effect on the total permittivity. Microwave-based animal detection systems have also used carcasses as test targets for sensor validation [33]. The carcass was stored under refrigeration and brought to room temperature (27 °C) before each measurement session.

### 2.5. Experimental Setup

**Figure 2:**
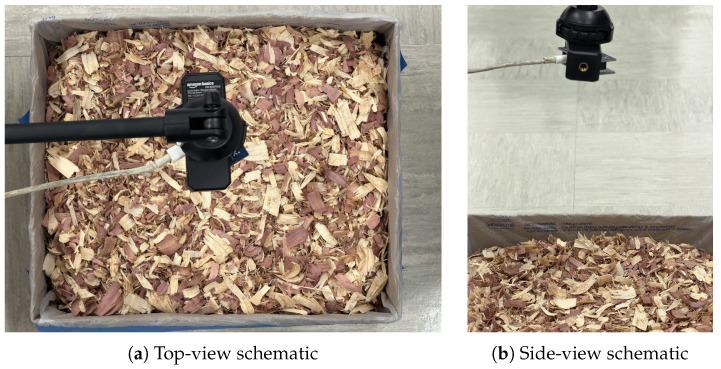
Experimental setup. (**a**) Top-view schematic showing the UWB radar module mounted on an adjustable-height tripod above the bedding container at three heights (30, 50, 70 cm). The container internal dimensions are 30 cm × 25 cm × 12 cm with 10 cm bedding depth. (**b**) Side-view schematic showing the estimated antenna footprint at each height.

The radar module was mounted on an adjustable-height tripod with the antenna pointing vertically downward toward the bedding surface. Three antenna heights were tested: 30, 50, and 70 cm above the top of the bedding. These heights were selected to represent a range of practical mounting distances in a poultry house environment (Figure 2). All measurements were conducted in a university laboratory in Tucson, AZ, USA. Ambient temperature was 27 °C and relative humidity was approximately 5%. This low value reflects the arid regional climate of southern Arizona. The possible influence of ambient humidity is discussed in Section 4.3.

Ground-truth LMC was determined by the oven-drying method following AOAC Method 930.15 [13]. A bedding sample of 450 ± 0.5 g was collected immediately after each UWB measurement, weighed on a digital balance (readability *d* = 0.5 g, precision 0.1 g), dried at 105 °C for 24 h, and re-weighed. Samples were not pre-dried before preparing target LMC levels. LMC was calculated on a wet basis (Equation (1)) as:

(1) $$\mathrm{LMC}=\frac{m_{\mathrm{wet}}-m_{\mathrm{dry}}}{m_{\mathrm{wet}}}\times 100\%$$

where $m_{\mathrm{wet}}$ and $m_{\mathrm{dry}}$ are the sample mass before and after drying, respectively.

A four-phase experimental program was designed to evaluate UWB moisture sensing under increasingly realistic poultry house conditions. Rather than extracting all features and designing the full model architecture from the outset, both the feature set and the model were developed progressively. At each phase, the model from the previous phase was first applied to the new data without retraining. The resulting degradation revealed a specific physical challenge, which motivated two responses: targeted feature expansion and architectural refinement. This progressive approach ensured that every feature had a clear physical justification and every architectural change addressed a demonstrated limitation. A total of 17 unique features were extracted from the CIR $h_{c}\left[k\right]$ of each virtual channel across the four phases (Table A1 and Table A2, and Appendix A). All per-channel features were extracted independently from each of the four virtual channels.

### 2.6. Phase 1: Baseline Signal–Moisture Characterization

**Experimental design.** The objective of Phase 1 was to establish the fundamental relationship between UWB signal features and bedding LMC in clean cedar shavings without any contamination or structural modification.

Bedding samples were prepared at six target LMC levels: 0, 10, 20, 30, 40, and 50% (wet basis). All moisture levels were set on a mass basis. For each target LMC, the required mass of distilled water was calculated from the initial dry mass of the bedding using the relation $m_{\mathrm{water}}=m_{\mathrm{dry}}\times {\mathrm{LMC}}_{\mathrm{target}}/\left(1-{\mathrm{LMC}}_{\mathrm{target}}\right)$. The dry bedding was weighed on a digital balance (readability *d* = 0.5 g), and the calculated water mass was added incrementally using a spray bottle. The wetted material was mixed by hand for 1 min and then allowed to equilibrate in a sealed bag for 10 min before measurement. The target LMC served only as the preparation guideline; the actual ground-truth LMC for each sample was determined independently by oven-drying (Section 2.5) and this verified value was used in all model training and evaluation. UWB signals were recorded at each of the three antenna heights. Five replicate measurements were performed per condition, yielding a total of 90 measurements (6 LMC levels × 3 heights × 5 replicates; Table 2).

**Feature extraction.** Figure 1b illustrates the raw CIR from which all features were derived. As moisture increased, the peak reflection amplitude grew and the peak range bin shifted to later indices due to reduced propagation velocity. Four per-channel features were extracted as direct indicators of permittivity contrast from moisture: Peak Amplitude ($A_{\mathrm{peak}}$), Peak Range Bin ($k_{\mathrm{peak}}$), Signal Energy (*E*), and Peak Phase ($\phi_{\mathrm{peak}}$). Applied to four virtual channels, these yielded a 16-dimensional feature vector and 10,800 frame-level samples (90 measurements × 120 frames).

**Model selection.** Five candidate regression algorithms were trained on the 16-dimensional feature set to select a base regressor: Ridge Regression (L2-regularized linear baseline), SVR with RBF kernel [34], LightGBM, XGBoost, and Gaussian Process Regression (GPR, RBF kernel with uncertainty estimates). All models were evaluated using stratified 5-fold cross-validation with 10 random repetitions. Hyperparameters were tuned via grid search within each training fold. As reported in Section 3.1, SVR achieved the best cross-validated performance and was adopted as the base regressor for all subsequent phases.

### 2.7. Phase 2: Manure Simulant Contamination

**Experimental design.** Phase 2 evaluated the effect of manure simulant contamination on UWB-based LMC estimation. The antenna was fixed at 50 cm, the height that yielded the best performance in Phase 1. In Phase 1, distilled water was used to bring the bedding to the target LMC. In Phase 2, the manure simulant described in Section 2.3 fully replaced water as the moisture source. Bedding samples were prepared at three LMC levels (10, 30, and 50%) using simulant only, homogenized before measurement. Five replicates were collected per LMC level, resulting in 15 measurements (3 LMC levels × 5 replicates).

**Feature expansion.** The ionic conductivity introduced by the manure simulant increases dielectric loss and signal dispersion. The four Phase 1 amplitude/energy features cannot isolate this effect from moisture-induced amplitude changes. To capture the dispersion and velocity shifts caused by ionic contamination, we introduced three delay-domain features: Phase Slope (Δϕ), Mean Excess Delay ($\bar{\tau }$), and RMS Delay Spread ($\sigma_{\tau }$). The Phase 1 SVR model was applied directly to Phase 2 data (1800 samples from 15 measurements) without retraining. The prediction errors quantified how much contamination degraded the baseline model. The three delay-domain features above were then appended, expanding the feature vector from 16 to 28 dimensions.

**Model update.** The SVR was retrained on the combined Phase 1 + 2 data (12,600 samples from 105 measurements).

### 2.8. Phase 3: Bedding Structural Variation

**Experimental design.** Phase 3 investigated how changes in bedding structure affect UWB signal propagation. During a typical grow-out cycle, litter transitions from a loose state to a compacted layer and eventually forms a hardened caked surface [11]. Three structural states were prepared to simulate this progression:

- **Loose:** freshly prepared shavings placed in the container without compression. The manure simulant was used as the sole moisture source, as in Phase 2.
- **Compacted:** shavings pressed uniformly using a weighted board for 12 h. This simulated the effect of prolonged bird traffic.
- **Caked:** compacted bedding with the top 1 cm further dried in an oven at 100 °C for 1 h to form a hardened surface crust overlying a moist interior.

Bulk density was measured for each structural state by weighing the container contents and dividing by the known container volume. Measurements were performed at three LMC levels (10, 30, and 50%) with three replicates per condition, yielding 27 measurements (3 structures × 3 LMC levels × 3 replicates).

**Feature expansion.** Compaction and caking alter bulk density, pore structure, and surface roughness. These changes reshape the specular reflection and multipath response of the CIR rather than its raw amplitude or delay. To characterize these waveform-shape changes, we introduced five features: Peak-to-Average Power Ratio (PAPR), Rise Time Slope ($S_{\mathrm{rise}}$), Skewness ($\gamma_{1}$), Kurtosis ($\beta_{2}$), and Full-Width at Half Maximum (FWHM). The Phase 1 + 2 SVR model was applied directly to Phase 3 data (3240 samples from 27 measurements) without retraining. The five waveform-shape features above were then appended, expanding from 28 to 48 dimensions.

**Mixture-of-experts architecture.** The single SVR regressor was replaced by a mixture-of-experts (MoE) architecture. A support vector classifier (SVC) with an RBF kernel was trained to classify three structural states (loose, compacted, caked), outputting class probabilities. Three SVR experts were trained, one per state, on the combined Phase 1 + 2 + 3 data (15,840 samples from 132 measurements). The final LMC prediction is a probability-weighted combination:

(2) $$\hat{\mathrm{LMC}}=\sum_{s=1}^{S}p_{s}\cdot {\hat{\mathrm{LMC}}}_{s}$$

where $p_{s}$ is the SVC probability for scene class *s* and ${\hat{\mathrm{LMC}}}_{s}$ is the prediction from the corresponding SVR expert. This soft-gating approach avoids catastrophic error from a single misclassification; when the SVC is uncertain, both experts contribute proportionally.

### 2.9. Phase 4: Stationary Bird Obstruction

**Experimental design.** Phase 4 was the central experiment of this study. In a commercial poultry house, birds are present on the litter at all times. Any ceiling-mounted moisture sensor must therefore operate reliably when a broiler body obstructs the measurement path. Phase 4 tested this capability under combined realistic conditions: manure contamination, structural variation, and stationary bird obstruction.

**Figure 3:**
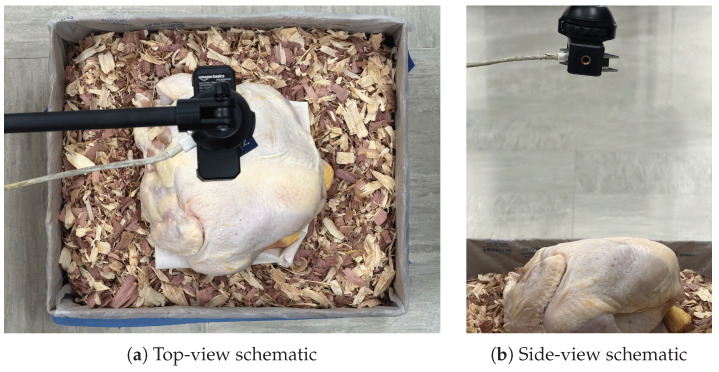
Phase 4 measurement configuration with carcass present. (**a**) Top-view schematic showing the carcass in centered, breast-down position directly beneath the antenna. (**b**) Side-view schematic showing maximum body contact with the bedding surface.

The broiler carcass described in Section 2.4 was used as a proxy for a live bird. The bedding was prepared using manure simulant as the sole moisture source, consistent with Phases 2 and 3. Two structural states were used: loose and caked (the two extremes from Phase 3). Two measurement configurations were tested:

1. **No carcass** (reference): unobstructed beam path with contaminated, structurally varied bedding. This provided a within-phase baseline.
2. **Carcass present:** carcass placed in a centered, breast-down position directly on the bedding surface beneath the antenna. This simulated a resting bird with maximum body contact, representing the worst-case obstruction scenario (Figure 3).

Measurements were performed at three LMC levels (10, 30, and 50%) with three replicates per condition. This yielded 36 measurements (2 carcass configurations × 2 structural states × 3 LMC levels × 3 replicates). The combined scenario allowed a direct assessment of whether the pipeline developed in the earlier phases could maintain acceptable accuracy when all confounding factors, including bird obstruction, were present simultaneously.

**Feature expansion.** The broiler body is a large, high-permittivity scatterer that introduces a dominant early reflection and suppresses high-frequency components of the echo. To capture this distinct scattering signature we introduced three per-channel spectral features (Spectral Centroid ($f_{c}$), Band Energy Ratio ($E_{LF/HF}$), and Energy Concentration Index (ECI)) and two MIMO cross-channel features (Inter-channel Cross-correlation ($\rho_{i,j}$) and Differential MIMO Phase ($\Delta \phi_{\mathrm{MIMO}}$)). Together, these five features characterize the spectral distortion and spatial diversity changes induced by body obstruction. The Phase 3 MoE pipeline was applied to Phase 4 data (4320 samples from 36 measurements) without retraining. The five new features above were then appended, expanding from 48 to 64 dimensions. Features 1–15 were computed per channel (60 dimensions); features 16–17 (MIMO cross-channel) were computed from two diagonal channel pairs (4 dimensions).

**Expanded mixture of experts.** The SVC gate was expanded from 3-class (structural state) to 6-class (3 structural states × 2 bird conditions), with six SVR experts. The same MoE formulation (Equation (2)) was applied with S=6. The SVC gate was fitted on all cumulative frames from Phases 1 to 4. Each SVR expert was fitted on the phase-specific partition of the same cumulative pool. The pipeline was evaluated in two modes: the Phase 1 + 2 + 3 MoE applied directly to Phase 4 data without retraining, and the full pipeline trained on cumulative Phase 1–4 data.

**Feature pre-screening.** The 64-dimensional vector is not consumed by a single monolithic regressor. The SVC gate routes each frame to one of six experts. Each expert operates on the feature subset most relevant to its regime. Before final training, highly correlated features (|ρ|>0.95) were collapsed to a single representative. Features with permutation importance below the mean null-shuffle level were dropped. The retained ranking is reported in Figure 11.

### 2.10. Summary of Experimental Conditions

**Figure 4:**
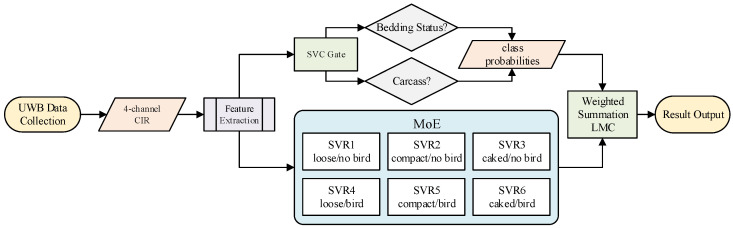
Architecture of the final SVC-gated mixture-of-experts pipeline. A 6-class support vector classifier routes the 64-dimensional feature vector to six scene-specific SVR experts via soft probabilistic gating (Equation (2)).

Across all four phases, the experimental program comprised 168 measurements and 20,160 frame-level samples (10,800 from Phase 1, 1800 from Phase 2, 3240 from Phase 3, and 4320 from Phase 4). Each phase used only the feature subset accumulated up to that point. Figure 4 illustrates the final pipeline architecture, and Table 3 summarizes the experimental matrix.

### 2.11. Statistical Analysis and Validation

**Model Evaluation Metrics.** Model performance was evaluated using three metrics: the coefficient of determination ($R^{2}$), root mean square error (RMSE), and mean absolute error (MAE). For the scene classifier, performance was assessed using overall classification accuracy, per-class precision, recall, and the confusion matrix.

**Cross-Validation Strategy.** All regression models were evaluated using stratified group 5-fold cross-validation. The grouping variable was the measurement ID. All 120 frames from a single 2 s measurement were assigned to the same fold. Stratification by LMC level ensured that each fold contained a representative proportion of the six moisture conditions. Measurement-level grouping prevented frame-level leakage between training and validation folds. Without grouping, metrics would be overly optimistic. The cross-validation was repeated 10 times with different random splits. Reported values are the mean ± standard deviation across all folds and repetitions. In Phase 1 (Section 2.6), all five candidate algorithms were compared under identical splits. The Wilcoxon signed-rank test assessed whether RMSE differences between the top-performing models were significant (p<0.05).

**Figure 5:**
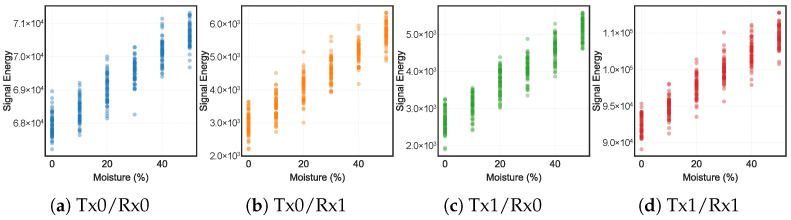
Signal energy (*E*) versus LMC for each virtual channel (clean cedar shavings, 50 cm antenna height; each point is one UWB frame). All four channels show a monotonic increase in *E* with LMC, confirming that the moisture-induced permittivity contrast is captured across all spatial diversity paths.

**Figure 6:**
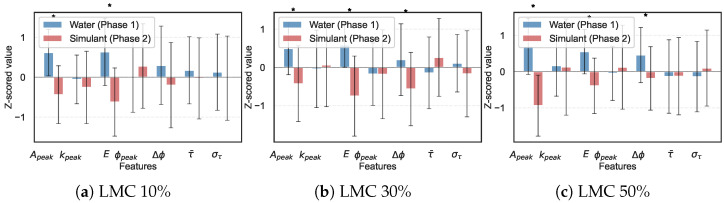
Comparison of extracted feature values between water-moistened (Phase 1, blue) and simulant-moistened (Phase 2, red) bedding at three matched LMC levels. Error bars show ±1 SD. Asterisks denote significant differences (p<0.05). Features are z-scored for visual comparability across different physical units.

**Statistical Comparison of Experimental Conditions.** All inferential comparisons used measurement-level summary statistics (mean feature value per measurement; *n* = 90, 15, 27, and 36 for Phases 1–4) rather than the 120 correlated frames per measurement. This ensured independence of the observations. The Shapiro–Wilk test checked normality. When normality was satisfied, one-way ANOVA was applied with Tukey HSD for pairwise comparisons. When normality was violated, the Kruskal–Wallis test was used with Dunn’s post hoc comparison. Throughout, α=0.05 with Bonferroni adjustment for multiple comparisons within a family. The tests applied to each variable were: moisture-dependent signal energy *E* across antenna heights (Figure 5, ANOVA + Tukey HSD); water vs. simulant feature shifts at matched LMC (Figure 6, Kruskal–Wallis + Dunn’s); structural-state feature distributions (Figure 9 and Table A5, ANOVA + Tukey HSD); and carcass vs. no-carcass attenuation (Table 9, paired Wilcoxon signed-rank on matched measurements).

**Threshold Detection Analysis.** In addition to continuous LMC estimation, a binary classification analysis was performed. Each measurement was classified as either “safe” (LMC <25%) or “at-risk” (LMC ≥25%) based on the estimated LMC value. This threshold corresponds to the LMC level above which footpad dermatitis risk increases substantially [3]. Binary classification accuracy, sensitivity, and specificity were reported for each model and scene type.

**Software.** All data processing, feature extraction, model training, and statistical analyses were performed in Python 3.10 using NumPy, SciPy, scikit-learn, and XGBoost. GPR was implemented using the scikit-learn GaussianProcessRegressor with an RBF kernel plus a white noise kernel.

## 3. Results

### 3.1. Baseline Signal–Moisture Characterization and Model Selection (Phase 1)

**Signal Feature Response to Moisture Content.**  Figure 5 shows the signal energy (*E*) extracted from clean cedar shavings at six LMC levels (0–50%) with the antenna at 50 cm height. *E* increased monotonically with LMC across all four virtual channels, consistent with the rising permittivity contrast between moist bedding ($\varepsilon_{r}$ increasing toward 20–30 at 50% LMC) and air. The vertical spread at each LMC level reflects frame-to-frame variation within each measurement.

As shown in Figure 5, the two co-located Tx/Rx pairs dominate the absolute reflected power. The two cross-pairs, with their wider Tx/Rx spatial baseline, carry lower absolute energy but show a larger relative rise across the 0–50% LMC range. The complementary behaviour of co-located and cross-pair channels motivates the use of all four virtual channels in the feature vector.

**Effect of Antenna Height.** Table 4 compares the best-performing model (SVR) at each antenna height. The 50 cm height yielded the lowest RMSE and was selected for all subsequent phases.

The 70 cm height produced the highest RMSE due to a reduced signal-to-noise ratio at greater distance. The 30 cm height did not outperform 50 cm despite the shorter range. This is attributed to edge effects from the small container: at 30 cm, the antenna beam footprint approached the container walls, and partial wall reflections contaminated the bedding signal. The 50 cm height provided the best trade-off between signal strength and beam footprint size within the laboratory container.

**Regression Model Comparison.** Table 5 compares the five candidate algorithms on Phase 1 data at 50 cm antenna height.

SVR with an RBF kernel achieved the lowest RMSE (2.48% LMC) and highest $R^{2}$ (0.97). The gap between SVR and Ridge Regression (RMSE 4.35% LMC) was statistically significant (Wilcoxon signed-rank test, p<0.001), confirming that the feature-LMC relationship contains nonlinear components that a linear model cannot capture. Even with only 16 features (4 per channel), the RBF kernel exploits nonlinear interactions between amplitude, energy, delay, and phase across multiple channels. GPR performed comparably to SVR (RMSE 2.61% LMC), but the difference was not statistically significant (p=0.12). SVR was selected as the base regressor for all subsequent steps because it achieved the lowest RMSE and is computationally efficient for deployment on embedded hardware.

**Figure 7:**
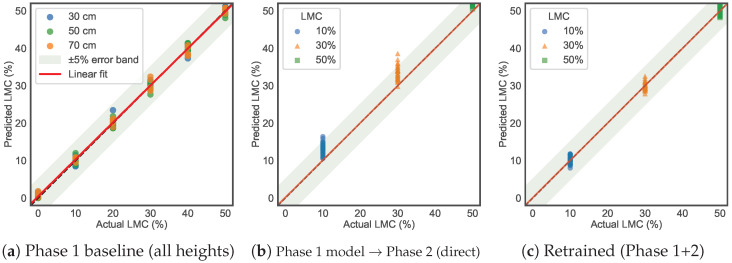
Predicted versus actual LMC across Phases 1 and 2. (**a**) Phase 1 baseline model (SVR with RBF kernel) across three antenna heights (blue = 30 cm, green = 50 cm, orange = 70 cm). Red line shows linear fit; shaded band spans ±5% LMC. (**b**) Phase 1 model applied directly to simulant-moistened Phase 2 data, showing systematic overestimation at 30% LMC. (**c**) Retrained SVR with expanded 28-dimensional feature set on combined Phase 1 + 2 data. In (**b**,**c**), marker shape indicates LMC level (∘ = 10%, Δ = 30%, □ = 50%).

Figure 7a shows the predicted versus actual LMC for the baseline model across all three antenna heights.

### 3.2. Effect of Manure Simulant Contamination (Phase 2)

**Feature Shift Due to Contamination.** Figure 6 compares the extracted feature values between water-moistened (Phase 1) and simulant-moistened (Phase 2) bedding at matched LMC levels. Signal energy and peak amplitude showed the largest shifts, consistent with the increased dielectric loss from ionic conductivity. Phase slope and mean excess delay also shifted, indicating that the simulant altered the effective propagation velocity. Peak range bin was largely unaffected by contamination.

**Progressive Model Degradation and Recovery.**  Table 6 shows the degradation when the Phase 1 model (16 features) was applied directly to contaminated data, and the recovery after feature expansion and retraining on combined data with 28 features Table 7.

As summarized in Table 6, applying the Phase 1 model directly to contaminated data yielded systematic overestimation of LMC. The ionic conductivity of the simulant attenuated the signal in a way the model interpreted as higher moisture. Feature expansion to 28 dimensions followed by retraining on the combined Phase 1 + 2 data recovered most of this degradation (see Table 6 for exact $R^{2}$, RMSE, and MAE values). Physically, the three added delay-domain features allowed the SVR to separate the dispersion effects of contamination from the amplitude changes caused by moisture alone. Figure 7 shows the predicted versus actual LMC before and after recovery.

### 3.3. Effect of Bedding Structural State (Phase 3)

**Bulk Density and Signal Characterization.**  Table 8 reports the measured bulk density for each structural state.

**Figure 8:**
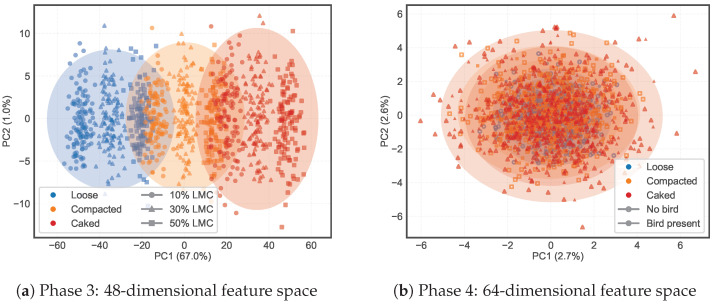
PCA projections of the feature space at two progressive stages. (**a**) Phase 3 (48 dimensions): three structural states form distinct clusters along PC1 (67.0% variance), with marker shape indicating LMC level. Ellipses show 95% confidence regions. (**b**) Phase 4 (64 dimensions): six scene classes (3 structural states × 2 bird conditions; open = no carcass, filled = carcass present).

Figure 8a shows the first two principal components of the 48-dimensional feature space for Phase 3 data. The three structural states formed distinct clusters along PC1, with the caked condition most separated due to its unique combination of high surface reflectivity and attenuated subsurface return. This clustering motivated the SVC-gated mixture-of-experts architecture: a single regressor cannot simultaneously fit the different feature-LMC relationships across these separated regions.

**Figure 9:**
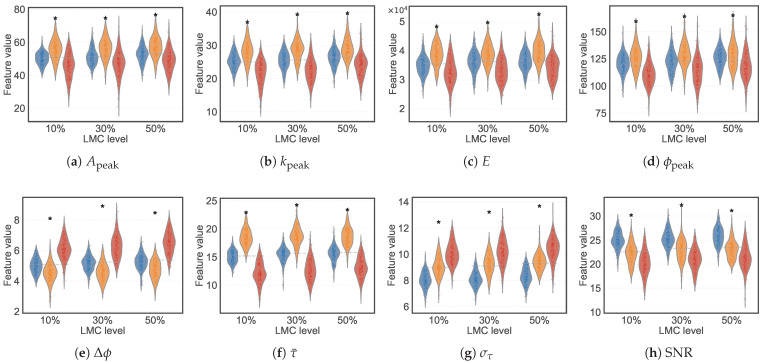
Violin plots of frame-level feature distributions across three structural states (blue = loose, orange = compacted, red = caked) at each LMC level (Phase 3). Individual data points are overlaid as jittered strips. Asterisks denote significant pairwise differences (Tukey HSD, p<0.05).

Figure 9 shows the frame-level feature distributions across the three structural states and indicates two complementary trends. Amplitude- and energy-domain features ($A_{\mathrm{peak}}$, $k_{\mathrm{peak}}$, *E*, $\phi_{\mathrm{peak}}$) rank compacted bedding highest and caked lowest. This ordering is consistent with stronger specular reflection from a denser surface and attenuation through a dry crust. Delay-domain features (Δϕ, $\sigma_{\tau }$) invert this ordering because the caked crust introduces an additional dielectric discontinuity and layered multipath that broaden the impulse response. Mean excess delay $\bar{\tau }$ provides the cleanest three-way separation: compaction shifts reflected energy to later delay bins. SNR decreases monotonically from loose to caked bedding. Exact medians and pairwise-significance markers are shown on the figure and are not repeated here.

ANOVA confirmed that structural state significantly affected the majority of per-channel features (Table A5 in Appendix B). The delay-domain features ($\bar{\tau }$, $\sigma_{\tau }$) showed the strongest effects because compaction and caking changed the spatial distribution of scatterers. The caked condition showed LMC-dependent feature variation despite the dry surface crust, confirming that UWB radar detects subsurface moisture beneath the crust. This is a concrete advantage over surface-only methods such as NIR.

**Progressive Model Degradation and Recovery.**

After expanding the feature set and switching to the SVC-gated MoE architecture, the pipeline handled clean, contaminated, and structurally varied bedding. The SVC structural state classifier achieved 94.2% overall accuracy on the three-class problem (loose, compacted, caked) under cross-validation. The soft gating ensured that misclassifications did not cause catastrophic regression errors. The cumulative RMSE across all 132 unobstructed measurements was 3.52% LMC. This established the performance baseline for clear line-of-sight conditions.

### 3.4. Moisture Estimation Through Stationary Broiler Body (Phase 4)

**Effect of Carcass on the Feature Space.** The presence of a stationary broiler carcass shifted the extracted features substantially. Figure 8b shows the first two principal components of the full 64-dimensional feature space across all six scene classes. Unlike the Phase 3 PCA projection (Figure 8a), where structural states separated clearly along PC1 (67.0% variance explained), the Phase 4 projection showed extensive overlap among classes in the first two components (cumulative variance only 5.3%). This indicates that the discriminative information is distributed across many dimensions rather than concentrated in the leading components, which justifies the use of the full 64-dimensional feature vector in the SVC classifier rather than a low-dimensional projection.

Table 9 quantifies the feature-level attenuation caused by the carcass relative to the no-carcass reference.

As quantified in Table 9, the centered breast-down position caused substantial attenuation of both peak amplitude and signal energy because the entire beam passed through the thickest part of the body. This worst-case configuration was selected deliberately. In a real house, birds alternate between resting (breast-down on litter), standing, and walking. Acceptable performance under the worst-case centered breast-down condition therefore implies at least comparable performance under other bird positions.

**Progressive Degradation and Recovery.** The Phase 3 MoE pipeline (48 features, 3-class SVC gate) was applied directly to Phase 4 data (4320 frame-level samples from 36 measurements). This produced the largest degradation observed in the study.

**Figure 10:**
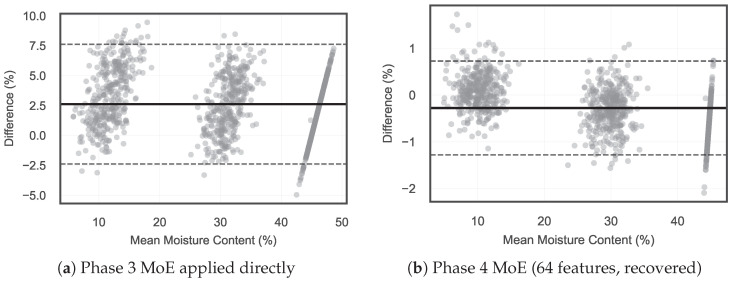
Bland–Altman agreement plots for Phase 4 data. (**a**) Phase 3 MoE applied directly (no bird awareness): positive mean bias and wide limits of agreement (±1.96 SD), reflecting systematic overestimation caused by carcass obstruction. (**b**) Phase 4 MoE with 64 features and 6-class gate: near-zero bias and tight limits of agreement, confirming that the bird-aware architecture recovered estimation accuracy.

As reported in Table 10, the Phase 3 MoE pipeline failed on bird-obstructed data. The carcass reflection dominated the feature space and pushed the feature vectors outside the training distribution. Expanding to 64 features and the 6-class MoE architecture substantially recovered accuracy across all structural sub-conditions. The exact $R^{2}$, RMSE, and MAE values are in Table 10 and are not duplicated here. Physically, the added spectral and MIMO cross-channel features enabled the SVC to distinguish bird-present from unobstructed scenes. The scene-specific SVR experts then adapted to the distinct feature distributions caused by body obstruction. Caked bedding under the carcass created three distinct dielectric layers (body, dry crust, moist interior), making it the hardest sub-condition. The system still retained acceptable accuracy on caked samples. Figure 10 presents the Bland–Altman agreement plots, confirming that the Phase 4 MoE eliminated the systematic bias observed when the Phase 3 pipeline was applied directly.

**Scene Classification Performance.** Table 11 shows the confusion matrix for the 6-class SVC scene classifier, which jointly identifies bedding structural state and bird presence. For clarity, the confusion matrix is collapsed to the bird-present vs. absent dimension.

**Figure 11:**
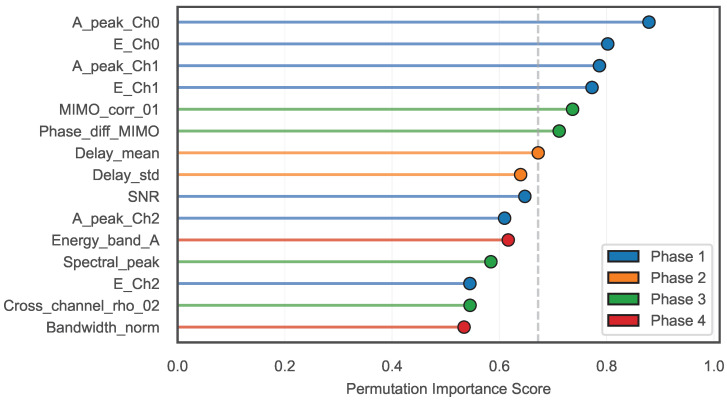
Feature importance ranking from the SVC scene classifier (top 15 of 64 features, permutation importance). Each lollipop is colored by originating phase (blue = Phase 1, orange = Phase 2, green = Phase 3, red = Phase 4). The vertical dashed line marks the mean importance across all 64 features. Amplitude-domain features (peak magnitude, energy) and cross-channel MIMO features ranked highest.

Figure 11 summarizes the permutation-importance ranking: amplitude/energy features dominate bird detection, whereas MIMO cross-channel features ($\rho_{i,j}$, $\Delta \phi_{\mathrm{MIMO}}$) contribute most to distinguishing structural states. The figure shows the retained subset after correlation pre-screening and permutation-importance thresholding (Section 2.9). Together, these results show that CIR-derived features alone are sufficient for scene classification, without any auxiliary sensor, and that soft-probability gating spreads predictions across experts when the classifier is uncertain.

**Threshold Detection at 25% LMC.** Table 12 reports the binary classification performance at the 25% LMC welfare threshold.

Even under combined conditions (Phase 4), the system maintained high sensitivity at the 25% LMC threshold. Sensitivity exceeded specificity in all phases. For a welfare monitoring application, this is the preferred trade-off. A false alarm (safe litter classified as at-risk) triggers an unnecessary investigation but causes no harm. A missed detection (at-risk litter classified as safe) allows wet conditions to persist and increases FPD risk.

### 3.5. Progressive Model Summary and Global Baseline Comparison

**Progressive Degradation and Recovery.** Table 13 is the central result table of this paper. It traces the model performance through each progressive development step, showing how each new confounding factor degraded accuracy and how each refinement recovered it.

**Figure 12:**
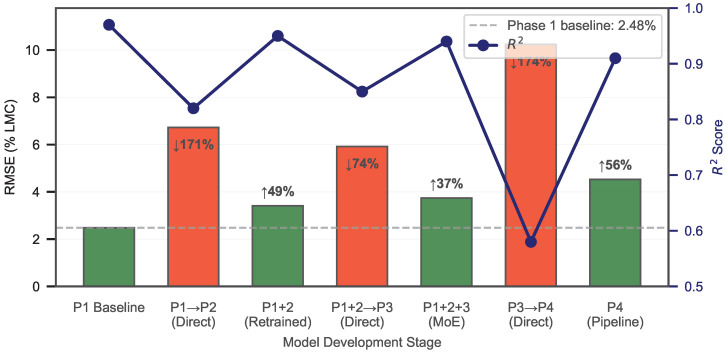
Progressive RMSE across model development stages. Green bars show post-recovery performance; red bars show degradation when a prior model is applied to new conditions. The horizontal gray dashed line marks the Phase 1 baseline (RMSE = 2.48%). Improvement percentages are annotated between stages. The navy line plot (right y-axis) shows $R^{2}$ evolution across the same stages, illustrating the degradation–recovery pattern across all four experimental phases. downward arrow indicates a decrease and upward arrow indicates an increase.

Figure 12 visualizes the degradation–recovery pattern across all four phases.

The progressive summary reveals a clear pattern. Each confounding factor (contamination, structural variation, stationary bird obstruction) degraded the model, and each degradation motivated targeted feature expansion and architectural refinement. Contamination was addressed by adding delay-domain features. Structural variation was addressed by adding waveform shape features and introducing the SVC-gated MoE architecture. Stationary bird obstruction required spectral, energy concentration, and MIMO cross-channel features, together with a 6-class SVC gate and six scene-specific SVR experts. The largest degradation occurred when the Phase 3 MoE was applied to bird-obstructed data, confirming that bird obstruction is the dominant challenge.

## 4. Discussion

### 4.1. Key Findings

The four-phase program suggests that UWB radar can estimate LMC under increasingly realistic poultry-house conditions. In Phase 1, a small per-channel feature set captured the permittivity contrast between water and dry shavings. The baseline SVR reached accuracy broadly comparable to contact-based capacitive sensors [15] and NIR reflectance [16] while remaining non-contact and volumetric.

Table 14 places these results alongside prior methods. Under clean, unobstructed conditions, UWB radar appears to match the accuracy of the listed techniques. The adaptive pipeline also maintained acceptable accuracy under combined contamination, structural variation, and bird obstruction. To our knowledge, no prior study has jointly tested these conditions. Capacitive probes require physical insertion and are sensitive to bulk-density changes [15]. NIR and hyperspectral methods are surface-only and cannot see through caked crusts or biological obstructions [17,18]. Microwave dielectric sensors require close proximity and density-dependent calibration [24]. These comparisons should be read as suggestive rather than definitive because they come from a laboratory proof-of-concept dataset.

**Summary.** Prior studies mainly evaluate performance under single or idealized conditions. The four-phase progressive design in this study quantifies and mitigates the compound effects of manure contamination, structural change, and bird obstruction. The results support the robustness of UWB radar for complex scenarios, pending field validation.

Each subsequent phase introduced a confounding factor that degraded the prior model. The mechanism of degradation matched the expected physics. Manure contamination in Phase 2 raised ionic conductivity and the imaginary permittivity, causing systematic overestimation. The three delay-domain features then allowed the SVR to separate contamination-induced dispersion from moisture-induced amplitude change. Structural variation in Phase 3 altered bulk density and produced layered reflections in caked bedding. The five waveform-shape features and the SVC-gated mixture-of-experts architecture mitigated this effect. The caked results support that UWB radar can penetrate a dry surface crust and detect subsurface moisture, which is not possible with surface-only methods such as NIR [18]. Stationary bird obstruction in Phase 4 caused the most severe degradation. The broiler carcass ($\varepsilon_{r}\approx 50$–55) [30,31] produced a dominant early reflection that shifted feature vectors outside the training range. The spectral and MIMO cross-channel features with the 6-class SVC gate and six scene-specific experts recovered accuracy. Exact $R^{2}$, RMSE, and MAE values are given in Table 6, Table 7, Table 10 and Table 13 and are not re-listed here. The contrast between the adaptive pipeline and a single global SVR suggests that scene-aware routing is essential, with the largest improvement seen on bird-obstructed data.

### 4.2. Practical Implications

The threshold detection results (Table 12) indicate that the system can distinguish litter below and above the 25% LMC welfare threshold [3]. Sensitivity is the more critical metric. A false alarm triggers an unnecessary investigation. A missed detection allows wet conditions to persist and increases the risk of footpad dermatitis.

A possible deployment is a ceiling-mounted UWB sensor array at about 2.5 m above the litter. The X7F202 antennas are approximately omnidirectional in elevation and 120 degrees wide at 3 dB in azimuth, so the sensor footprint is set by moisture SNR rather than by beam width. A simple link budget based on the module’s 13.6 m receive window and 36 dB input dynamic range places the useful sensing radius at several meters. Taking a conservative 4 m effective footprint (about 12 $m^{2}$ per sensor), a 150 m × 15 m house requires on the order of 40 sensors at roughly 7.5 m by 5 m spacing. This density is sufficient for a zonal ventilation controller. At a bare module price of about US$30, the hardware cost is approximately US$1200 per house, excluding the embedded computer, cabling, power, and integration labor. Bulk procurement would likely reduce the per-unit cost. The X7F202 consumes less than 100 µW in standby and the pipeline runs in under 1 ms on an embedded computer, which is competitive with robotic platforms and hyperspectral camera systems [12]. UWB radar combines contactless sensing, volumetric penetration through caked surfaces, and operation through biological obstructions. The SVC gate detects bird presence and routes each measurement to the appropriate expert, so bird obstruction is handled automatically. A spatial moisture map can be fed to the ventilation controller for closed-loop management.

### 4.3. Limitations and Future Work

Several limitations must be acknowledged. First, the 120 frames per measurement are temporally correlated. Cross-validation splits were grouped at the measurement level, and inferential tests used per-measurement summary statistics (Section 2.11). Reported metrics reflect between-measurement generalization. Second, 64 features were trained on 168 independent measurement setups. Overfitting therefore remains a risk for future field deployments. Correlation pre-screening and a permutation-importance threshold were applied to prune redundant features, and group-aware cross-validation was used throughout (Section 2.9 and Section 2.11). Larger independent datasets with nested feature selection will be needed to re-validate the retained subset in the field. Third, all experiments used a 30 × 25 cm container at 5% RH (Section 2.5). Commercial houses have higher humidity, ammonia, dust, and metallic multipath. Fourth, only cedar shavings were tested, and the synthetic simulant does not reproduce microbial decomposition or spatial heterogeneity. Fifth, the refrigerated carcass was held at 27 °C, whereas a live bird has a core temperature near 41 °C. This 14 °C offset shifts the Debye relaxation of free water by roughly 2%/°C and lowers $\varepsilon^{\prime \prime }$ at 7.875 GHz [30]. Features sensitive to attenuation and phase slope may need light in situ recalibration under live-bird conditions. Full retraining is unlikely to be required. The carcass was also stationary, so movement, posture change, respiration, and thermoregulation are not captured. Finally, the 6-class scene classifier was evaluated with a single bird. Commercial conditions include multiple birds, partial overlap, and equipment in the beam.

The most critical next step is field validation with live birds in a research poultry house. Such validation must assess long-term stability and reliability under dynamic bird activity and realistic environmental conditions. Point-accuracy metrics alone are not sufficient. A longitudinal study over a full grow-out cycle (35–42 days) should report regression accuracy, SVC scene-misclassification rate, the fraction of time the sensor is occluded, the failure rate, and the effect of humidity and temperature on calibration. Further work should extend the scene classifier to handle multiple birds, partial overlap, moving birds, and equipment in the beam. Transfer learning could reduce calibration effort across bedding materials. Sensor fusion with temperature, humidity, and ammonia readings may further improve LMC estimation. Online learning with periodic oven-drying spot checks could adapt the model to house-specific conditions without complete retraining.

## 5. Conclusions

This study investigated UWB impulse radar as a non-contact method for estimating poultry bedding moisture content under laboratory conditions. A four-phase experimental program progressively isolated the effects of moisture level, manure contamination, bedding structural variation, and stationary broiler-body obstruction. Phases 1–3 indicate that a progressive feature-engineering approach combined with an SVC-gated mixture-of-experts architecture can maintain acceptable accuracy across clean, contaminated, and structurally varied bedding without a bird. The results also suggest that UWB radar can detect subsurface moisture beneath a dry crust. Surface-only methods such as NIR spectroscopy cannot.

Phase 4 shows, for the first time to our knowledge, that UWB radar can estimate LMC when a stationary broiler body obstructs the beam. Without mitigation, bird obstruction caused substantial model failure. Accuracy was recovered by expanding the feature set and by expanding the SVC gate to six classes (three structural states by two bird conditions) with six scene-specific SVR experts. The pipeline also detected bird presence from the radar signal alone with high accuracy. At the 25% LMC welfare threshold, it achieved sensitivity consistent with use for binary welfare alerting. Exact values are given in Table 12 and Table 13. Taken together, these laboratory findings support UWB radar as a potentially viable candidate for non-contact litter moisture monitoring in precision poultry farming. Field validation with live birds is the essential next step. Such validation should assess long-term stability and failure modes, not only point accuracy.

## Figures and Tables

**Table 1:** Radar module configuration parameters. SDK parameter names are listed for reproducibility.

Parameter	SDK Name	Value	Derived Quantity
Frame rate	FPS	60	–
Pulse period	PulsePeriod	4	PRF = 32.81 MHz
Pulses per iteration	PulsesPerIteration	35	2240 pulses/frame
Iterations per frame	IterationsPerFrame	64	
Mini-frames per pulse	MframesPerPulse	4	–
Transmit power	TxPower	4 (max)	2.5 dBm peak
Interleaved frames	InterleavedFrames	5	12 sets/s
Tx channel sequence	TxChannelSequence	[0, 1]	Alternating Tx0/Tx1
Rx mask sequence	RxMaskSequence	[3, 3]	Both Rx active (0b11)
DC removal	DCRemoval	false	Removed in software

**Table 2:** Phase 1 experimental matrix: baseline signal–moisture characterization.

Factor	Levels	Values
Moisture content	6	0, 10, 20, 30, 40, 50%
Antenna height	3	30, 50, 70 cm
Replicates	5	
Total measurements		90

**Table 3:** Summary of the experimental program across all four phases.

Phase	Factors Varied	Conditions	Replicates	Total
1: Baseline	6 LMC × 3 heights	18	5	90
2: Manure	3 LMC	3	5	15
3: Structure	3 LMC × 3 structural states	9	3	27
4: Stationary bird	3 LMC × 2 structures × 2 carcass	12	3	36
Grand total				168

**Table 4:** Effect of antenna height on LMC estimation accuracy (Phase 1, SVR with RBF kernel). Values are mean ± standard deviation from 10× stratified 5-fold cross-validation.

Antenna Height (cm)	$R^{2}$	RMSE (% LMC)	MAE (% LMC)
30	0.95 ± 0.02	3.24 ± 0.41	2.56 ± 0.33
50	0.97 ± 0.01	2.48 ± 0.31	1.89 ± 0.24
70	0.93 ± 0.02	3.92 ± 0.48	3.05 ± 0.38

**Table 5:** Comparison of five regression algorithms for LMC estimation on Phase 1 data (clean bedding, 50 cm height, 16-dimensional feature vector, n=10,800 frame-level samples). Values are mean ± standard deviation from 10× stratified 5-fold cross-validation. The best result in each column is shown in bold.

Algorithm	$R^{2}$	RMSE (% LMC)	MAE (% LMC)
Ridge Regression	0.92 ± 0.03	4.35 ± 0.52	3.41 ± 0.45
**SVR (RBF)**	**0.97** ± **0.01**	**2.48** ± **0.31**	**1.89** ± **0.24**
LightGBM	0.95 ± 0.02	3.12 ± 0.38	2.45 ± 0.30
XGBoost	0.96 ± 0.02	2.87 ± 0.35	2.21 ± 0.28
GPR (RBF)	0.96 ± 0.02	2.61 ± 0.33	2.02 ± 0.26

**Table 6:** Progressive model performance on Phase 2 data. “Phase 1 model (direct)” used 16 features and was trained only on clean bedding. “Retrained (Phase 1 + 2)” used the expanded 28-dimensional feature set and was trained on combined data (n=12,600 frame-level samples from 105 measurements) downward arrow indicates a decrease.

Model	$R^{2}$	RMSE (% LMC)	MAE (% LMC)
Phase 1 model (direct)	0.82	6.73	5.21
Retrained (Phase 1 + 2)	0.95	3.41	2.68
RMSE improvement	–	3.32% LMC (↓49%)	–

**Table 7:** Progressive model performance on Phase 3 data. “Phase 1 + 2 model (direct)” used 28 features and was not exposed to structural variation during training. “MoE (Phase 1 + 2 + 3)” used the expanded 48-dimensional feature set with SVC-gated mixture of experts. downward arrow indicates a decrease.

Model	$R^{2}$	RMSE (% LMC)	MAE (% LMC)
Phase 1 + 2 SVR (direct, 28 feat.)	0.85	5.92	4.63
MoE (Phase 1 + 2 + 3, 48 feat.)	0.94	3.74	2.91
RMSE improvement	–	2.18% LMC (↓37%)	–

**Table 8:** Measured bulk density of cedar shaving bedding under three structural states at 30% LMC.

Structural State	Bulk Density (g/cm^3^)	Relative to Loose (%)
Loose	0.08 ± 0.01	100
Compacted	0.14 ± 0.02	175
Caked	0.16 ± 0.02	200

**Table 9:** Signal attenuation caused by centered breast-down carcass obstruction relative to the no-carcass reference. Values averaged across LMC levels, structural states, and replicates.

Configuration	$A_{\mathrm{peak}}$ Reduction (dB)	*E* Reduction (dB)
No carcass (reference)	0.0	0.0
Centered, breast-down	−8.7	−10.2

**Table 10:** Progressive model performance on Phase 4 data. “Phase 3 MoE (direct)” used the 48-feature, 3-class MoE pipeline. “Phase 4 MoE (64 feat.)” used the expanded 64-dimensional feature set with the 6-class SVC gate and six SVR experts.

Model	Structure	$R^{2}$	RMSE (% LMC)	MAE (% LMC)
Phase 3 MoE applied directly (48 features, 3-class gate):				
Phase 3 MoE (direct)	All	0.58	10.24	8.17
Phase 3 MoE (direct)	Loose	0.67	8.53	6.74
Phase 3 MoE (direct)	Caked	0.48	12.15	9.83
Phase 4 MoE (64 features, 6-class gate, 6 experts):				
Phase 4 MoE	All	0.91	4.53	3.52
Phase 4 MoE	Loose	0.94	3.82	2.97
Phase 4 MoE	Caked	0.87	5.38	4.21

**Table 11:** Confusion matrix for the SVC scene classifier (collapsed to bird detection). Evaluated by 5-fold cross-validation on all Phase 1–4 data.

	Predicted: No Bird	Predicted: Bird
Actual: No bird	17,856	144
Actual: Bird	97	2063

Bird detection accuracy: 98.8%. Full 6-class accuracy: 92.1%.

**Table 12:** Binary classification performance at the 25% LMC threshold (safe vs. at-risk) across experimental phases.

Phase	Accuracy (%)	Sensitivity (%)	Specificity (%)
Phase 1 (clean)	98.9	100.0	97.8
Phase 2 (contaminated)	94.4	94.4	94.4
Phase 3 (structural)	92.6	93.3	91.7
Phase 4 (stationary bird)	89.8	91.7	87.5

**Table 13:** Progressive model development summary. Each row shows the model performance when applied to a specific test condition. “Feat.” indicates feature dimensionality. In the final row, “Phase 4 (MoE)” denotes the pipeline trained on cumulative data from Phases 1 to 4. The SVC gate is fitted on all cumulative frames, and each SVR expert is fitted on its phase partition. “Phase 4 (CV)” denotes the Phase 4 cross-validation partition used for evaluation. The table reads top to bottom as baseline, degradation, recovery with feature expansion, degradation, recovery with MoE, catastrophic failure, recovery with full pipeline. downward arrow indicates a decrease and upward arrow indicates an increase.

Training Data	Test Data	Feat.	$R^{2}$	RMSE	Narrative
Phase 1 (SVR)	Phase 1 (CV)	16	0.97	2.48	Baseline
Phase 1 (SVR)	Phase 2	16	0.82	6.73	↓ Contamination
Phase 1 + 2 (SVR)	Phase 2 (CV)	28	0.95	3.41	↑ +3 features
Phase 1 + 2 (SVR)	Phase 3	28	0.85	5.92	↓ Structure
Phase 1 + 2 + 3 (MoE)	Phase 3 (CV)	48	0.94	3.74	↑ +5 feat. + MoE
Phase 3 MoE	Phase 4	48	0.58	10.24	⇓ **Bird (failure)**
Phase 4 (MoE)	Phase 4 (CV)	64	0.91	4.53	⇑ +5 feat. + 6-class

**Table 14:** Comparison of litter and substrate moisture sensing methods. “Caked” indicates whether the method can measure subsurface moisture beneath a dry crust. “Birds” indicates whether the method was tested with a biological obstruction in the measurement path. RMSE values are in % moisture content.

Study	Technology	Contact	$R^{2}$	RMSE	Caked	Birds
Xiong et al. [15]	Capacitive probe	Yes	0.90–0.94	—	No	No
Xiong et al. [15]	NIR reflectance	No	0.97–0.99	—	No	No
Reeves & Van Kessel [16]	NIR spectroscopy	No	0.95	1.0	No	No
Mowrer et al. [35]	NIR spectroscopy	No	0.996	—	No	No
Cockerill et al. [17]	Hyperspectral	No	0.92–0.97	1.0–1.6	No	No
Trabelsi & Nelson [24]	Microwave dielectric	Yes	—	<1.0	No	No
Huisman et al. [21]	GPR (soil)	No	0.90–0.95	1.5–3.0	Yes	No
This work (Phase 1)	UWB radar	No	0.97	2.48	—	—
This work (Phase 3)	UWB radar + MoE	No	0.94	3.74	Yes	—
This work (Phase 4)	UWB radar + MoE	No	0.91	4.53	Yes	Yes

## Data Availability

The data presented in this study are available on request.

## Abbreviations

The following abbreviations are used in this manuscript:

UWB	Ultra-Wideband
LMC	Litter Moisture Content
FPD	Footpad Dermatitis
PLF	Precision Livestock Farming
CIR	Channel Impulse Response
PDP	Power Delay Profile
ToF	Time-of-Flight
SNR	Signal-to-Noise Ratio
RMSE	Root Mean Square Error
NIR	Near-Infrared
SVC	Support Vector Classifier
SVR	Support Vector Regression
MoE	Mixture of Experts
MIMO	Multiple-Input Multiple-Output
PAPR	Peak-to-Average Power Ratio
FWHM	Full Width at Half Maximum
ECI	Energy Concentration Index

## Appendix A. Feature Definitions

Table A1 summarizes the progressive feature expansion across the four experimental phases, and Table A2 provides the complete definitions, mathematical formulations, and physical motivations for all 17 unique features extracted from the UWB channel impulse response $h_{c}\left[k\right]$, where *c* denotes the virtual channel and *k* the range bin index.

**Table A1:** Progressive feature expansion across experimental phases.

Phase	New Features	Total Dim.	Features Added and Motivation
1: Baseline	4 per ch.	16	Peak Amplitude, Peak Range Bin, Signal Energy, Peak Phase. Direct indicators of permittivity contrast from moisture.
2: Manure	3 per ch.	28	Phase Slope, Mean Excess Delay, RMS Delay Spread. Capture dispersion and velocity changes from ionic contamination.
3: Structure	5 per ch.	48	PAPR, Rise Time Slope, Skewness, Kurtosis, FWHM. Capture waveform distortion from compaction and caking.
4: Stationary bird	3 per ch. + 2 MIMO	64	Spectral Centroid, Band Energy Ratio, ECI, Inter-channel Cross-correlation, Differential MIMO Phase. Capture spectral attenuation and spatial diversity from body obstruction.

**Table A2:** Complete feature definitions. Features 1–15 are computed per channel (×4 channels = 60 dimensions). Features 16–17 are MIMO cross-channel features computed from diagonal channel pairs (×2 pairs = 4 dimensions). Total: 64 dimensions.

#	Feature	Symbol	Definition and Physical Motivation
Phase 1: Baseline moisture indicators (4 per channel)			
1	Peak amplitude	$A_{\mathrm{peak}}$	$\max_{k}\left|h_{c}\left[k\right]\right|$. Higher moisture raises the permittivity contrast at the air-bedding interface, increasing reflection amplitude.
2	Peak range bin	$k_{\mathrm{peak}}$	$\arg\max_{k}\left|h_{c}\left[k\right]\right|$. Proxy for time-of-flight; higher permittivity reduces propagation velocity, shifting the peak to a later bin.
3	Signal energy	*E*	$\sum_{k=k_{1}}^{k_{2}}{\left|h_{c}\left[k\right]\right|}^{2}$. Integrates total reflected power over the bedding window; less sensitive to noise than peak value.
4	Peak phase	$\phi_{\mathrm{peak}}$	$∠h_{c}\left[k_{\mathrm{peak}}\right]$. Phase shift of the reflected signal at the peak bin; complementary to amplitude as a permittivity indicator.
Phase 2: Delay-domain and propagation features (3 per channel)			
5	Phase slope	Δϕ	Linear slope of unwrapped phase across the bedding reflection window. Sensitive to propagation velocity changes from ionic contamination.
6	Mean excess delay	$\bar{\tau }$	$\sum_{k}k|h_{c}{\left[k\right]|}^{2}/\sum_{k}{\left|h_{c}\left[k\right]\right|}^{2}$. First moment of the PDP; contamination shifts the energy centroid to later delays.
7	RMS delay spread	$\sigma_{\tau }$	$\sqrt{\sum_{k}{\left(k-\bar{\tau }\right)}^{2}|h_{c}\left[k\right]{|}^{2}/\sum_{k}{\left|h_{c}\left[k\right]\right|}^{2}}$. Second central moment of PDP; contamination broadens the pulse through frequency-dependent absorption.
Phase 3: Waveform shape features (5 per channel)			
8	PAPR	–	$10\log_{10}(A_{\mathrm{peak}}^{2}/\mathrm{mean}(|h_{c}{\left[k\right]|}^{2}))$. Distinguishes specular reflection from compacted surfaces (high) versus diffuse scattering in loose litter (low).
9	Rise time slope	$S_{\mathrm{rise}}$	Slope of leading edge from 10% to 90% of $A_{\mathrm{peak}}$. Caked litter blunts the onset due to the layered crust.
10	Waveform skewness	$\gamma_{1}$	Third standardized moment of PDP. Caked bedding produces positive skew from trailing subsurface return.
11	Waveform kurtosis	$\beta_{2}$	Fourth standardized moment of PDP. Loose litter yields a sharp peak (high); compacted or caked media produce flatter profiles (low).
12	FWHM	–	Width of main reflection peak at half-maximum power. Compaction narrows the peak; caking and high moisture broaden it.
Phase 4: Spectral and MIMO features (3 per channel + 2 cross-channel)			
13	Spectral centroid	$f_{c}$	$\sum_{f}f|H_{c}{\left(f\right)|}^{2}/\sum_{f}{\left|H_{c}\left(f\right)\right|}^{2}$. Muscle tissue attenuates higher frequencies, shifting the centroid downward when a bird is present.
14	Band energy ratio	$E_{LF/HF}$	Ratio of lower-half to upper-half spectral energy. Higher ratio indicates high-frequency attenuation from body tissue.
15	Energy concentration index	ECI	Percentage of total energy within a narrow window around $k_{\mathrm{peak}}$. Bird obstruction scatters energy into additional bins, decreasing ECI.
16	Inter-channel cross-corr.	$\rho_{i,j}$	Max normalized cross-correlation between diagonal channel pairs. Bird body attenuates paths unequally, reducing inter-channel similarity.
17	Differential MIMO phase	$\Delta \phi_{\mathrm{MIMO}}$	Difference in $\phi_{\mathrm{peak}}$ between symmetric channels. Cancels common-mode noise; preserves asymmetric spatial obstruction.

## Appendix B. Statistical Analysis Tables

Table A3 reports Pearson correlation coefficients between signal features and LMC for Phase 1 data. Table A4 summarizes the ANOVA results for the effect of liquid type (Phase 1 vs. Phase 2). Table A5 presents the ANOVA results for the effect of structural state (Phase 3).

**Table A3:** Pearson correlation coefficients (*r*) between selected per-channel signal features and bedding moisture content for Phase 1 data (n=10,800 frame-level samples). Bold values indicate |r|>0.8.

Feature	Symbol	*r* vs. LMC
Peak amplitude	$A_{\mathrm{peak}}$	**0.93**
Peak range bin	$k_{\mathrm{peak}}$	−0.41
Signal energy	*E*	**0.95**
Mean excess delay	$\bar{\tau }$	0.72
RMS delay spread	$\sigma_{\tau }$	0.68
Peak phase	$\phi_{\mathrm{peak}}$	**−0.85**
Phase slope	Δϕ	−0.78

**Table A4:** One-way ANOVA results for the effect of liquid type (water vs. manure simulant) on selected per-channel signal features (Phase 1 vs. Phase 2, matched LMC levels). Significant effects (p<0.05) are shown in bold.

Feature	*F*-Statistic	*p*-Value	Significant?
$A_{\mathrm{peak}}$	8.42	**0.002**	**Yes**
$k_{\mathrm{peak}}$	1.87	0.162	No
*E*	11.56	<**0.001**	**Yes**
$\bar{\tau }$	3.24	**0.038**	**Yes**
$\sigma_{\tau }$	2.91	0.051	No
$\phi_{\mathrm{peak}}$	6.73	**0.005**	**Yes**
Δϕ	5.18	**0.011**	**Yes**

**Table A5:** One-way ANOVA results for the effect of structural state on selected per-channel signal features (Phase 3, n=3240 frame-level samples, pooled across LMC levels after LMC-detrending). Significant effects (p<0.05) are shown in bold.

Feature	*F*-Statistic	*p*-Value	Significant?
$A_{\mathrm{peak}}$	6.15	**0.008**	**Yes**
$k_{\mathrm{peak}}$	2.34	0.118	No
*E*	9.87	<**0.001**	**Yes**
$\bar{\tau }$	12.41	<**0.001**	**Yes**
$\sigma_{\tau }$	14.28	<**0.001**	**Yes**
$\phi_{\mathrm{peak}}$	4.52	**0.022**	**Yes**
Δϕ	7.83	**0.003**	**Yes**

## Appendix C. Bulk Electrical Conductivity of the Manure Simulant

This appendix provides the detailed derivation summarized in Section 2.3. The simulant contained peat moss (40%), bentonite clay (15%), water (43%), and sodium chloride (2%) by mass.

In a 100 g batch, 2 g of NaCl was dissolved in 43 g of water. The pore-water NaCl concentration was therefore 2/43≈46.5 g/L, or about 0.80 M at 25 °C. A 0.80 M NaCl solution has a free-solution conductivity of approximately 7.0 S/m [36].

Not all of this water was free to conduct current. Peat moss immobilizes water by hydrogen bonding to cellulose and hemicellulose. Bentonite binds water in its interlayer galleries. Kellner and Halldórsson [37] reported that the bound water fraction in *Sphagnum* peat can exceed 60% at low moisture content. A bound fraction of $f_{\mathrm{bound}}=0.60$ was assumed, giving a free fraction of 0.40.

The effective conductivity of a saturated porous medium is described by Archie’s law:

(A1) $$\sigma_{b}=\sigma_{w}\;\phi^{m}S_{w}^{n},$$

where $\sigma_{b}$ is the bulk conductivity, $\sigma_{w}$ is the pore-fluid conductivity, ϕ is the porosity, $S_{w}$ is the water saturation, *m* is the cementation exponent, and *n* is the saturation exponent. For a saturated paste with ϕ=0.5 and m=1.5, the term $\phi^{m}=0.5^{1.5}\approx 0.354$. Applying this correction to the effective free-water conductivity of 0.40×7.0=2.80 S/m yields

$$\sigma_{b}\approx 0.354\times 2.80=1.0\;S/m\;\;\left(10\;\mathrm{dS}/m\right).$$

Varying *m* from 1.3 to 1.7 and $f_{\mathrm{bound}}$ from 0.5 to 0.7 gives a plausible range of 0.6 to 1.5 S/m for the saturated paste.

When the simulant was used as the sole moisture source in Phases 2–4, the bedding-simulant mixture EC decreased roughly linearly with simulant mass fraction. At 50% LMC, the estimated mixture EC fell in the single-digit dS/m range. This matched the 4.5–7.2 dS/m reported for commercial broiler litter [28,29]. The ionic conductivity raises the imaginary component of the complex permittivity $\varepsilon^{\prime \prime }$, attenuates the radar signal, and reduces the reflected power from the bedding layer [25].
